# Thoracoscopic Repair of Long-Gap Esophageal Atresia Using the Thoracoscopic Internal Traction Technique: A Challenging Feat

**DOI:** 10.7759/cureus.72484

**Published:** 2024-10-27

**Authors:** Piotr R Więckowski, Joanna Łysak, Marek Wolski

**Affiliations:** 1 Department of Paediatric Surgery, Medical University of Warsaw, Warsaw, POL

**Keywords:** esophageal atresia, esophageal atresia repair, esophageal lengthening, internal traction technique, long-gap esophageal atresia, thoracoscopic internal traction, video-assisted thoracoscopic surgery (vats)

## Abstract

Long-gap esophageal atresia (LGEA) is an abnormality in the development of the esophagus resulting in the disruption of the continuity of the esophageal lumen with no feasible primary repair due to the "long gap" between two esophageal stumps. There is controversy regarding both the precise definition and the treatment protocol of this congenital condition. Methods such as delayed primary repair, open external traction, thoracoscopic external traction, and thoracoscopic internal traction were used in the treatment of patients with varied outcomes. Thoracoscopic internal traction was employed in the treatment of the described patient, who had a good functional outcome. Complications were managed conservatively with no need for additional surgeries. The four-week waiting period between both stages of the surgery made it possible to extubate the patient, forgo the postoperative need for paralysis, and reduce the period of sedation to the minimum. We believe that minimally invasive surgery can be used in the treatment of LGEA with good outcomes, provided the surgeon has sufficient experience.

## Introduction

Long-gap esophageal atresia (LGEA) is a congenital abnormality that presents as a disrupted continuity of the esophagus. There is no agreement on the definition of the "long gap," with various proposals [1,2] ranging from the "inability to repair by primary anastomosis" [3] to specific distances between esophageal stumps [4] or the lack of gas in the abdomen [5]. There is no consensus on whether the definition of LGEA should be confined to specific types as per Gross classification [2]. In the case of our case report, a definition of the "inability to repair by primary anastomosis" was employed.

Esophageal atresia can be suspected in pregnancy with signs such as polyhydramnios and a small or absent gastric bubble [3]. Postnatally, esophageal atresia should be suspected in an infant not tolerating oral feeding, presenting excessive drooling, or respiratory symptoms related to feeding. The diagnosis can be confirmed by the inability to pass the orogastric tube beyond 10 cm [3]. There is now an agreement to avoid performing a radiocontrast study prior to the repair, as the possible complications can be fatal [5]. There also is no consensus on the best management method for LGEA; many authors experienced success with delayed primary anastomosis [6], which is now favored by APSA (American Pediatric Surgical Association) and ERNICA (European Reference Network for Rare Inherited and Congenital (digestive and gastrointestinal) Anomalies) [2,5]. However, novel esophageal lengthening techniques such as external traction with thoracoscopic placement of traction sutures [7] and internal traction [1,8-13] are being employed with great outcomes and few complications. This work was previously presented at the Polish Society of Pediatric Surgeons Congress in September 2022.

## Case presentation

A premature (33 weeks and four days gestation) male infant was delivered vaginally, weighing 2310 g, with no signs of respiratory distress. The patient was admitted to the Surgical Clinic of the Medical University of Warsaw's Pediatric Hospital due to a suspicion of esophageal atresia, signs of which, such as polyhydramnios, were noticed during pregnancy. The pregnancy was complicated by cervical insufficiency and was treated with pessary since the 15th week of gestation. Polyhydramnios was treated with two amnioreduction procedures. Moreover, genetic testing performed during pregnancy confirmed 3q26.33 deletion in the *SOX2* gene. Mutations and deletions in this gene may cause a variety of symptoms encompassing esophageal atresia, anophthalmia, malformations of the external genitourinary tract, and neurodevelopmental problems [14]. It should be noted that the patient described in this study was examined multiple times by an ophthalmologist who described no significant abnormalities in the eye. The patient's genitourinary tract was normal and described as "age-appropriate."

**Figure 1:**
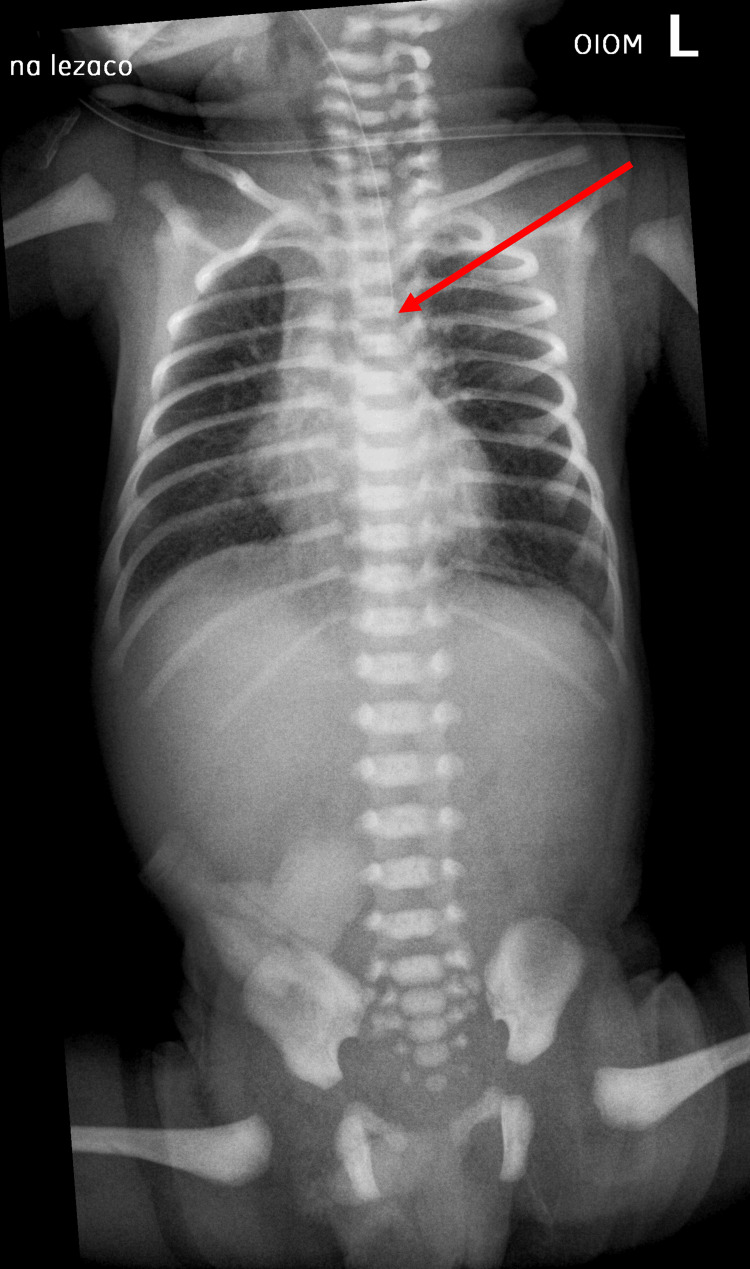
The "babygram" X-ray reveals a gasless stomach and intestines. An orogastric tube is inserted. The red arrow points to the end of the orogastric tube, which could not be passed further down.

Due to such suspicion, a "babygram" whole torso X-ray study was scheduled to confirm it. Esophageal atresia was confirmed by the inability to pass an orogastric tube, as shown in the radiograph (Figure 1). The patient received total parenteral nutrition.

To avoid extended total parenteral nutrition, a surgical gastrostomy was performed, and feeding was initiated. During endoscopy through both the oral cavity and gastrostomy, the atresia was confirmed once again with a notably short distal esophageal pouch, only 10-15 mm long. After a four-week waiting period, the patient was qualified for thoracoscopic repair of LGEA.

**Figure 2:**
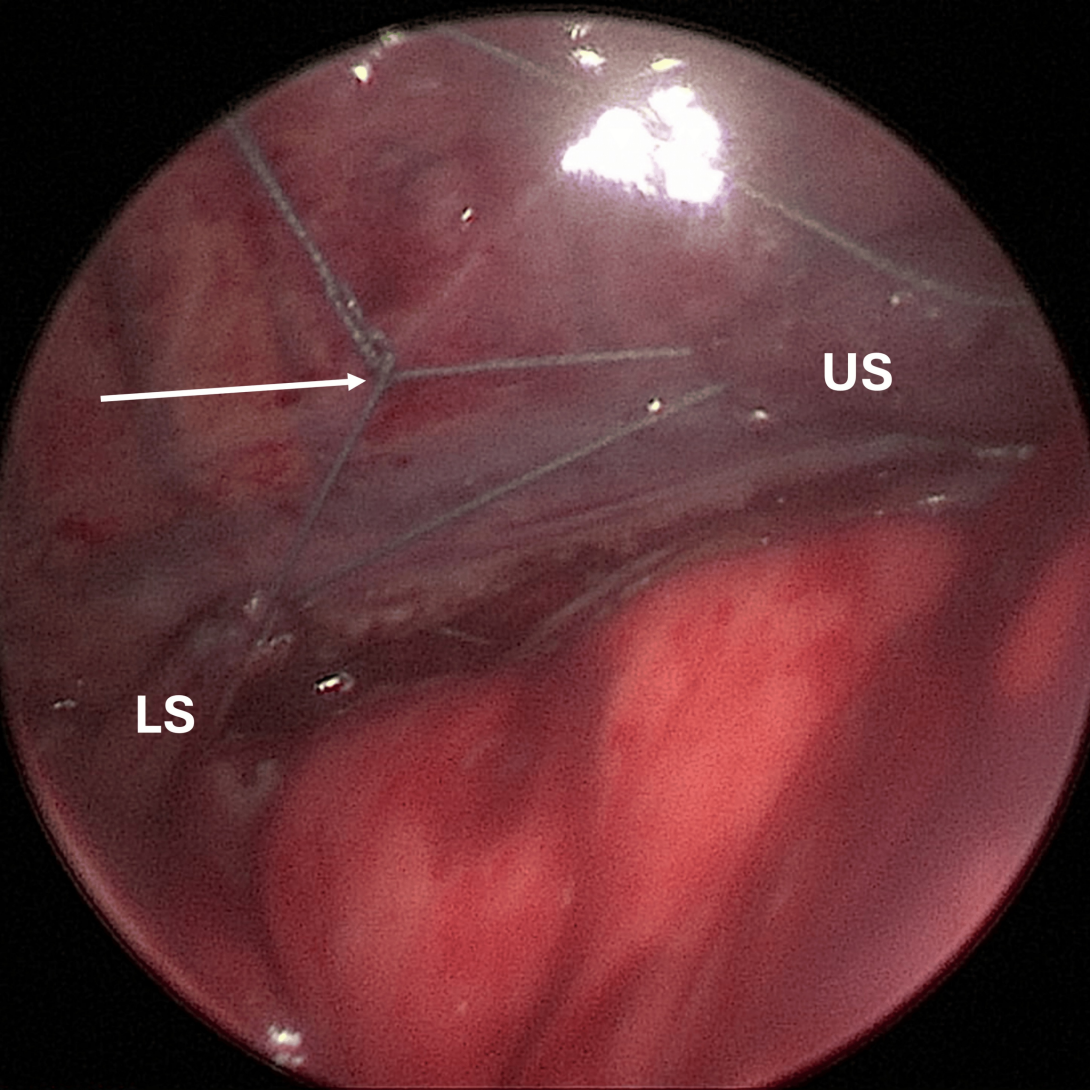
Intraoperative capture. Esophageal stumps are sutured together using a sliding knot under moderate tension. The long gap between the stumps should be noted. The white arrow points to the sliding knot used to achieve tension between the stumps. US: upper stump; LS: lower stump.

An incision was made in the posterior axillary line below the scapula, and a 3.5 mm trocar was inserted. Another 3.5 mm trocar was inserted below the first one, and a 5 mm trocar was placed above and laterally to the first one. The upper pouch was located with the help of a nasogastric tube and dissected in the thoracic inlet. The smaller lower pouch was located in the vagus nerve line below the diaphragm. The length of both pouches was assessed as not sufficient for an anastomosis. An internal traction suture reinforced with a clip to prevent leakage was placed on each pouch to bring them together and left them at moderate tension using a sliding knot (Figure 2). A chest drain was placed, and the trocar wounds were closed.

A month later, the anastomosis surgery was performed.

**Figure 3:**
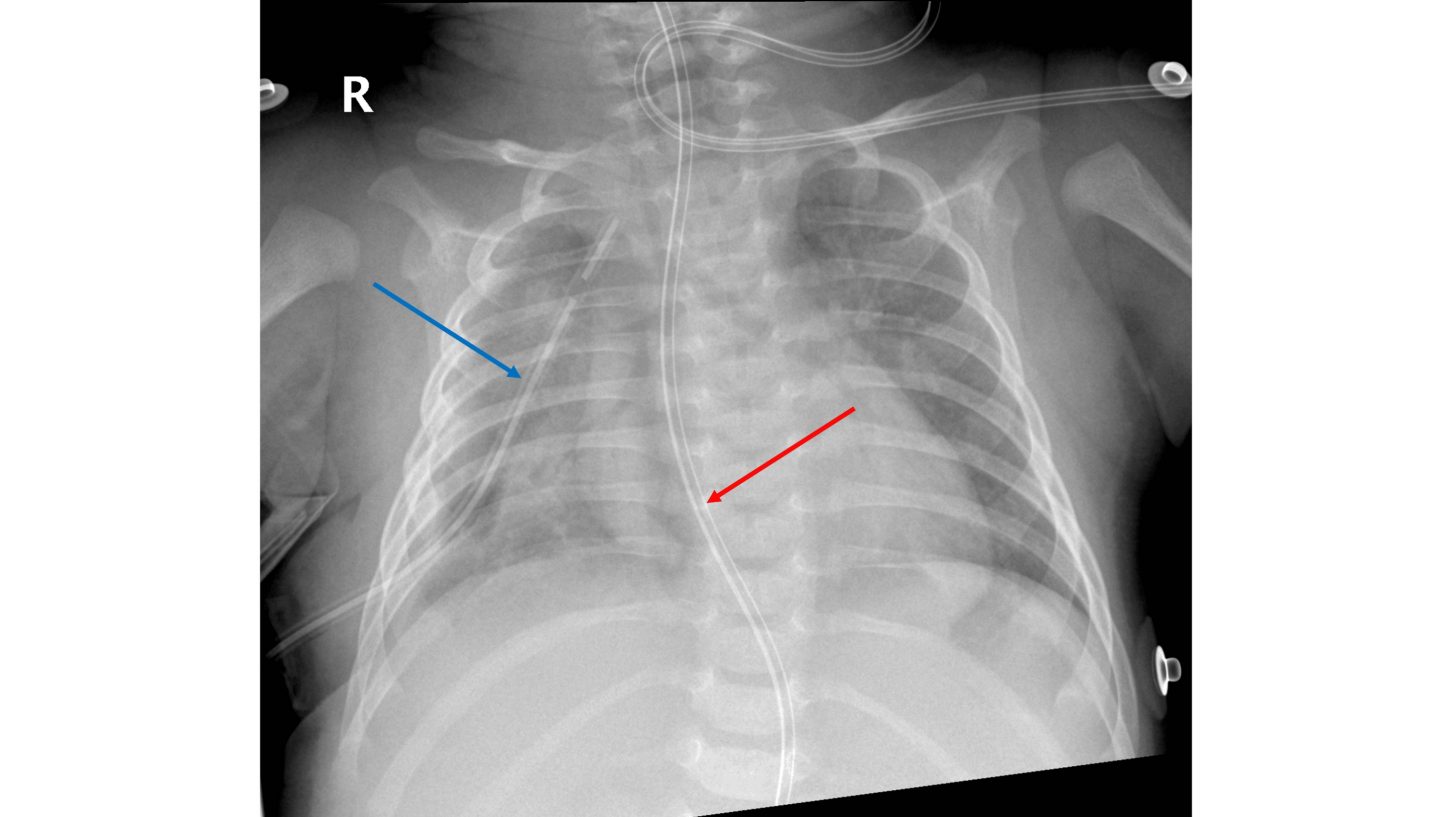
Postoperative chest X-ray showing the successful passage of the nasogastric tube along with a correct placement of the chest drain. The red arrow points to the nasogastric tube, and the blue arrow points to the chest drain.

The patient was placed on the stomach with the right part of the body elevated. Incisions were made in the scars from the previous surgery, and trocars were inserted. Both pouches were dissected, and the mediastinal adhesions were removed. The length of the pouches was assessed as sufficient for an anastomosis. The pouches were opened, and the anastomosis was created using single interrupted sutures. The nasogastric tube was left inside the anastomosis. A chest drain was placed, and the thoracic wall was closed. A chest X-ray performed after surgery shows the successful passage of the nasogastric tube and the correct placement of a chest drain (Figure 3).

**Figure 4:**
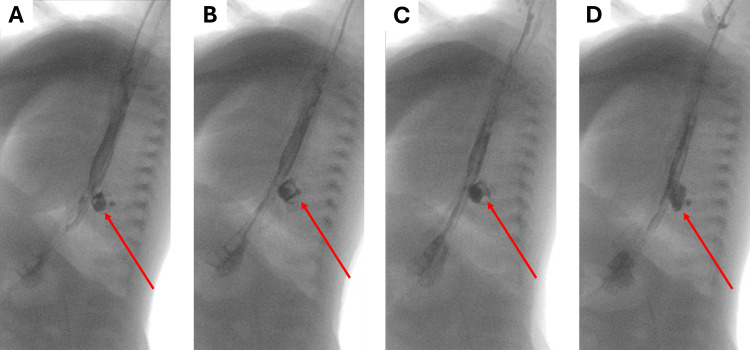
The radiocontrast evaluation shows signs of anastomotic leakage one week after the final surgery. (A) Initial X-ray taken upon an introduction of the contrast medium into the esophagus; (B) second X-ray taken after a waiting period; (C) third X-ray taken after a waiting period; (D) fourth X-ray taken after a waiting period. The time difference between each panel was at the discretion of the radiographer performing the evaluation. The red arrows point to the signs of radiocontrast leakage from the site of the esophageal anastomosis.

A week later, an upper gastrointestinal study was performed, in which signs of radiocontrast fluid leakage were found (Figure 4).

**Figure 5:**
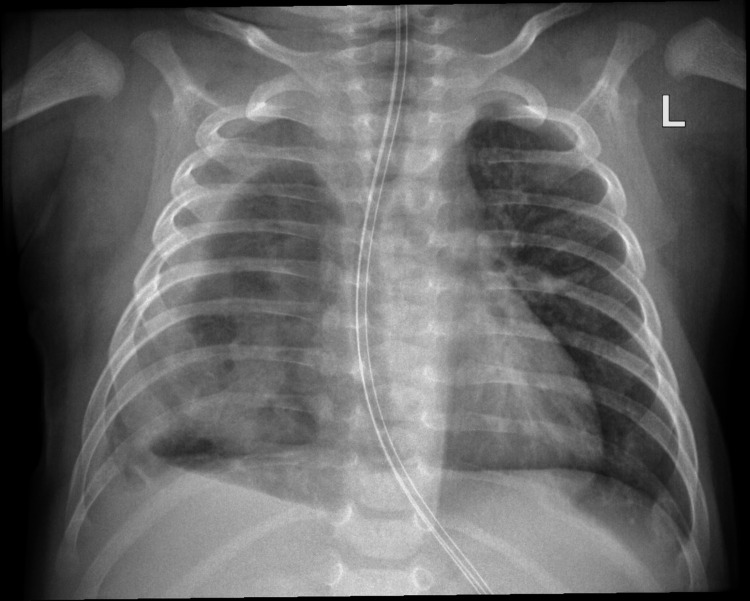
A chest X-ray taken a week after surgery showed fluid levels in the right pleural cavity along with pneumothorax and atelectasis of the right lung.

The patient developed signs of pneumonia. A chest X-ray performed a day later revealed pneumothorax and 9 mm of fluid in the right pleural cavity alongside atelectasis of the right lung (Figure 5).

**Figure 6:**
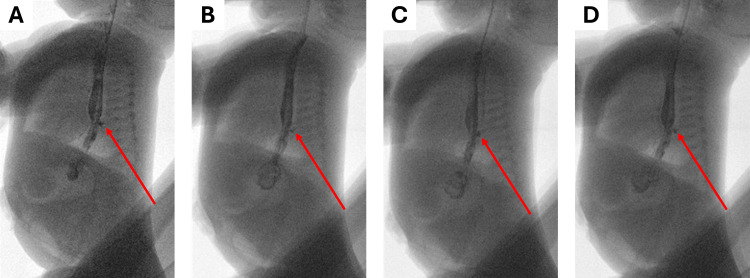
A radiocontrast evaluation performed two weeks after the final surgery showed a decreasing anastomotic leakage. (A) Initial X-ray taken upon an introduction of the contrast medium into the esophagus; (B) second X-ray taken after a waiting period; (C) third X-ray taken after a waiting period; (D) fourth X-ray taken after a waiting period. The time difference between each panel was at the discretion of the radiographer performing the evaluation. The red arrows point to the sites of radiocontrast leakage from the esophageal anastomosis.

A chest drain was inserted. A few days later, a repeat study showed a decrease in the radiocontrast leakage with a stricture forming in the place of the anastomosis (Figure 6).

**Figure 7:**
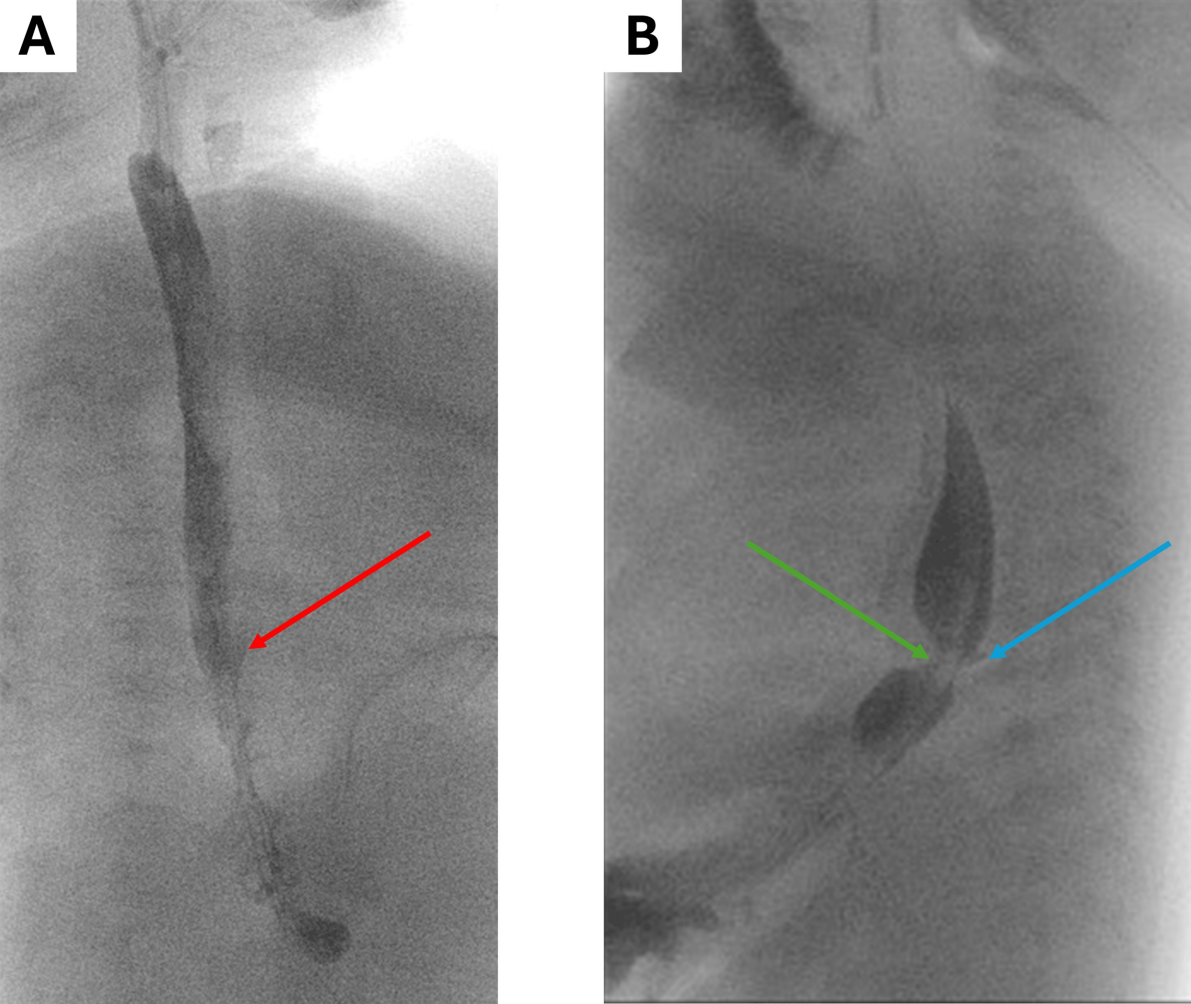
(A) A radiocontrast study performed three weeks after surgery revealed linear and barely visible anastomotic leakage. (B) A radiocontrast study performed four weeks after surgery revealed minimal anastomotic leakage. The stricture of the esophageal anastomosis is evident. (A) The red arrow points to the linear anastomotic leakage; (B) the blue arrow points to the barely visible anastomotic leakage, and the green arrow points to the visible stricture at the site of the anastomosis.

Two additional evaluations performed in weekly intervals showed decreasing leakage, which was eventually described as linear and barely visible (Figure 7).

Twenty-three days after the surgery, the nasogastric tube was removed, and oral alimentation was introduced. After a week of oral alimentation tolerance, the gastrostomy tube was removed, and the patient was discharged.

Although the second procedure was performed with low tension, four esophageal dilation procedures were required. The first procedure was performed using a 5F Savary-Gilliard dilator (Cook Medical, Bloomington, USA), and the second to fourth procedures were performed using angioplasty balloons of increasing diameter, arriving at 7.65 mm diameter. The last two were performed after esophageal blockage, most likely caused by reduced motility, as the esophageal diameter was adequate. A final dilation performed 21 months after the second surgery resulted in an esophageal diameter of 12 mm.

## Discussion

Various authors consider a thoracoscopic approach to the repair of LGEA viable, although due to significant operative difficulty, it should be reserved for specialized centers with a high volume of cases. As Patkowski emphasized in his seminar in 2023 [13], this concentration of cases provides better outcomes.

The authors adopted the delayed anastomosis technique, which requires waiting for the esophagus to grow. This technique provides only a gastrostomy tube for feeding before the surgical intervention within the thorax. Although gastrostomy is not required in the management of LGEA [9], this patient underwent this procedure to avoid risks of deep venous catheter placement for longer periods, such as infection and thrombosis. This enabled a safer and more physiological way of providing the patient with nutrition.

Elongation of the esophagus in cases of LGEA is still a controversial topic, with various success rates dependent on the method used, team experience, and neonatal comorbidities.

As recommended by the APSA Outcomes and Evidence-Based Practice Committee, the standard approach is to perform a delayed primary anastomosis [2]. This approach often results in the natural lengthening of both esophageal ends and allows for an anastomosis. Performing such anastomoses under tension can require the infant to be paralyzed during the initial period after the surgery [11,15], which creates additional difficulties and potential complications [16]. Moreover, some patients do not experience sufficient esophageal growth during the waiting period and require a different type of intervention, be it traction or esophageal replacement [2,3].

The van der Zee technique of thoracoscopic suture placement followed by external traction [7] provides good outcomes without the need for paralysis compared to the method developed by Foker [4]. However, this method still requires intubation and sedation during the four to six days between the first and second stages of the procedure. Intubation and sedation contribute to significant morbidities, such as ventilator-acquired pneumonia (VAP) [16], and may impair the neurological development of the infant [17].

The thoracoscopic internal traction technique developed by Patkowski and described in detail in his various works [1,8-11,13] enables the surgeon to perform a totally internal mode of esophageal traction achieved by applying sliding knots to the esophageal pouches and securing them by clips. In the time between surgeries, the infant is usually sedated and intubated but does not require paralysis. During the second surgery, adhesions are removed, and esophageal pouches are anastomosed if possible; if not, additional thoracoscopic surgeries providing increasing amounts of tension can be performed. This approach has numerous advantages, such as the exclusive use of minimally invasive surgery, the potential to avoid gastrostomies if performed promptly, and the mitigation of infection risks associated with externally placed traction. Many of these factors contributed to the decision to use internal traction combined with the delayed final anastomosis, as described by Rothenberg [18], in our patient.

In our case, the period between the surgeries was one month. This allowed the patient to grow in size and mass and allowed the traction to work for a longer period. Moreover, the patient was extubated and weaned off sedation in a standard approach, mitigating the need for a lengthy neonatal intensive care unit stay and the risk of neurodevelopmental problems. This is in contrast to the method described by Patkowski [11], who recommended a shorter waiting period between surgeries (three to five days), during which the patient is intubated, ventilated, and sedated. As the patient's nutritional needs were provided for using gastrostomy, our team was not constrained by the time between the two stages of the procedure, which is, unfortunately, the case when using total parenteral nutrition.

The anastomotic leak encountered while treating this patient was managed conservatively due to the infant's overall good condition and the resolution of symptoms with chest drainage.

Despite performing the anastomosis without extensive tension, the patient still required esophageal dilations. This is consistent with other authors' experiences [9,12]. The greater number of esophageal dilations needed in our patient can be attributed to the anastomotic leakage that exacerbated the stricture.

The fifth and sixth esophageal dilation procedures were indicated because the patient swallowed chunks of food extensive in size (the largest was 2 cm × 3 cm). The dilation performed during these procedures was prophylactic in nature and allowed the surgeons to dilate the esophagus to the final diameter of 12 mm. We suspect that those incidents of esophageal blockage were caused not by the small diameter of the stricture but by the intrinsically impaired esophageal motility associated with LGEA.

## Conclusions

This case report proves the validity of the thoracoscopic internal traction technique. The patient was treated with an excellent final outcome, with complications that resolved with conservative treatment, eliminating the necessity for additional surgeries. This method allowed us to mitigate many intrinsic risks associated with other esophageal lengthening methods. Although technically challenging, thoracoscopic repair of esophageal atresia is feasible and safe if performed by a sufficiently experienced team.
